# Key Lessons from COVID-19: A Narrative Review Describing Qatar’s Multifactorial Approach in Executing a Vaccination Campaign

**DOI:** 10.3390/vaccines11050953

**Published:** 2023-05-06

**Authors:** Soha Albayat, Muna Almaslamani, Hamad Alromaihi, Hayat Khogali, Jesha Mundodan, Jean Joury, Hammam Haridy

**Affiliations:** 1 Ministry of Public Health, Doha P.O. Box 42, Qatar; 2 Communicable Disease Center, Hamad Medical Corporation, Doha P.O. Box 3050, Qatar; 3 Pfizer Gulf FZ LLC, Dubai P.O. Box 502749, United Arab Emirates

**Keywords:** BNT162b2, mRNA-1273, Qatar, vaccination campaign, mass vaccination program, COVID-19 vaccines

## Abstract

Widespread vaccination programs have been implemented in many countries to curtail the COVID-19 pandemic, with varying success and challenges. To better understand the successes and challenges of the global COVID-19 response in the face of emerging new variants and epidemiologic data, we discuss how Qatar engaged the healthcare sector, governmental bodies, and the populace to combat COVID-19, with a focus on the country’s vaccination strategy. This narrative provides the history and timeline of the Qatar COVID-19 vaccination campaign; factors that helped the vaccination campaign and the transferable lessons learned are discussed. Details regarding how Qatar responded to challenges, such as vaccine hesitancy and mitigation of misinformation, are highlighted. Qatar was one of the first countries to procure the BNT162b2 (Comirnaty^®^; Pfizer-BioNTech, Pfizer Inc., New York, NY, USA) and mRNA-1273 (Spikevax^®^; Moderna, Cambridge, MA, USA) COVID-19 vaccines. A relatively high vaccination rate and low case mortality rate (0.14% as of 4 January 2023) was observed in Qatar compared with other countries (global case mortality rate, 1.02%). Learnings will be carried forward as a basis for addressing this evolving pandemic and any future national emergencies in Qatar.

## 1. Introduction

Since being declared a global pandemic by the World Health Organization (WHO), more than 755 million cases and >6.8 million deaths worldwide have been attributed to coronavirus disease 2019 (COVID-19) [1,2]. Efforts to mitigate spread have included physical distancing, quarantine measures, and stay-at-home orders. Although these efforts had varied success, they were also associated with detrimental effects on economic, social, and mental well-being [3,4]. The implementation of widespread vaccination programs was required to curtail the pandemic, with various countries enacting vaccination and with varying success. These responses also evolved temporally because of new variants, emerging epidemiologic data, and information from past measures and from other countries. Indeed, reviews documenting country- or region-specific approaches to the COVID-19 pandemic [5,6,7,8,9,10,11,12] provide an excellent opportunity to learn from the experiences gained throughout the crisis and to ensure the more efficient management of future viral outbreaks, thus limiting their toll [13].

Qatar is a small country with distinct population demographics. In 2020, the population of Qatar was approximately 2.8 million; 72% of the population were male, and 83% were 15–64 years old [14]. The population consists largely of non-national workers (94%) in economically productive age groups. Craft and manual workers often work in development projects and live in crowded, shared accommodations [15]. The average life expectancy in Qatar is 79 years for men and 82 years for women [16]. Although the population is generally healthy, high body mass index, high fasting plasma glucose, and dietary risks are leading risk factors contributing to disease burden [17].

In the past decade, Qatar has developed a modern healthcare system, with both public and private sectors [18,19,20]. Improvements included the establishment of several new hospitals and primary care and well-being centers [18]. With relatively high per capita health expenditure among the Gulf Cooperative Council (GCC) states, good quality healthcare is provided to all Qatari nationals and residents at greatly subsidized rates [19]. An existing national immunization program provides protection against 15 vaccine-preventable diseases in line with locally and globally identified disease trends [21].

To better understand the challenges faced and successes achieved during the evolving COVID-19 pandemic, this narrative review outlines how Qatar engaged the healthcare sector, governmental bodies, and the populace to combat the impact of COVID-19, with a focus on the country’s vaccination strategy. A critical appraisal of the campaign and transferable lessons are provided.

## 2. Search Strategy

To identify publications relevant to Qatar’s COVID-19 response and real-world data regarding the use of vaccines in Qatar, a literature search was conducted through PubMed for COVID-19- or SARS-CoV-2-specific publications through 4 January 2023, reporting data from Qatar. Narrative reviews and articles in languages other than English were excluded. Data extracted from the studies included approved vaccines; vaccination programs and recommendations; and vaccine uptake, safety, and effectiveness. Ad hoc searching of additional sources, including grey literature, was also conducted.

## 3. Response to Emergence of COVID-19 in Qatar

In response to the emergence of COVID-19, Qatar increased testing capacity to approximately 4000 people per day [22]. Contact tracing, social distancing, quarantines (including cordon sanitaire), and the closing of nonessential businesses were implemented [23]. Formal travel restrictions were enacted by 31 March 2020, with restrictions on incoming flights preventing nearly all visitors and residents from entering Qatar [22]. Travel out of the country diminished because of a global decrease in the number of flights worldwide and the inability of essential workers to travel or take leave outside of emergency situations.

**Figure 1:**
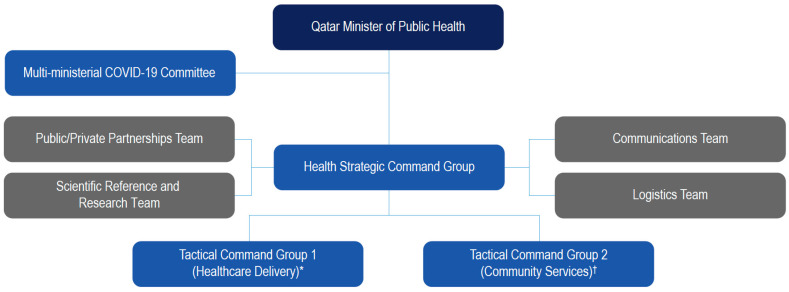
Governance structure of the Qatar National Response Program to COVID-19. * Responsibilities include delivery of healthcare across public and private facilities and accommodation for positive cases. ^†^ Responsibilities include quarantine, contact tracking and tracing, port of entry screening, and lockdown areas. (Reproduced from Al Khal A, Al-Kaabi S, Checketts RJ. Qatar’s response to COVID-19 pandemic. *Heart Views* 2020, *21*, 129–132 [18]).

In March 2020, Qatar began developing a National Response Plan (NRP) to the COVID-19 pandemic, which laid out a strategic framework detailing actions that were already completed, those that were in progress, and those that remained to be implemented [24]. The overall goal was to protect the health, well-being, and prosperity of Qatar’s citizens and residents by preparing for, monitoring, responding to, and recovering from COVID-19 outbreaks. As part of the NRP, a cross-functional government/agency team was assembled to support the health sector; maintain essential and critical services; and ensure that public confidence in the government, its agencies, and its processes was maintained (Figure 1) [24]. Qatar’s planned and organized governance structure enabled rapid and informed decision-making [18].

With COVID-19 spreading rapidly among migrant workers, access to healthcare in Qatar became a challenge for these communities [23]. Qatar dedicated some hospitals to care for patients with COVID-19, while still providing routine care in non–COVID-19 hospitals [18]. In particular, Qatar provided dedicated health centers to treat migrant workers with COVID-19 close to where they reside [25]. Approximately 4000 acute and intensive care beds were available for COVID-19–related care, although full acute care bed capacity was not reached, even during the pandemic’s first wave. Adequate provision of hospital beds, along with rigid infection control measures and an ensured supply of personal protective equipment, allowed for consistently high clinical staffing levels. Other key measures implemented in Qatar included urgent allocation of isolation and quarantine facilities; chest radiography, electrocardiogram, and phlebotomy services; early identification and treatment of pneumonia cases; rapid and early expanded tele-health and telemedicine services; increased focus on public health messaging; and the creation of multilingual tools to maximize outreach.

The Ministry of Interior and the Ministry of Public Health (MOPH) launched the Ehteraz app for smartphones in May 2020 [26]. This mandatory application tracked viral transmission chains and provided users with accurate information.

Although vaccines were not available at the time of writing the NRP, the vaccination campaign comprised a significant vision for the future and Qatar’s holistic approach [24]. As described in the following sections, the availability of vaccines and the implementation of a wide-reaching vaccination campaign, which was the largest ever implemented in the country, have been critical components of Qatar’s pandemic response.

## 4. The Qatar COVID-19 Vaccination Campaign

In December 2020, BNT162b2 (Comirnaty^®^; Pfizer-BioNTech. Pfizer Inc., New York, NY, USA) became the first vaccine for which findings for protection against COVID-19 were reported [27]. The phase 2/3 study in >43,000 individuals aged ≥16 years showed 95% vaccine efficacy against COVID-19 after receipt of 2 doses. Subsequently, another mRNA COVID-19 vaccine, mRNA-1273 (Spikevax^®^; Moderna, Cambridge, MA, USA), reported findings from a phase 3 study in >30,000 participants ≥18 years old [28]. After administration of two mRNA-1273 doses, vaccine efficacy for the prevention of symptomatic SARS-CoV-2 infection was 94%.

Qatar was one of the first countries to procure COVID-19 vaccines and to start a COVID-19 vaccination campaign [29]. After approval for COVID-19 vaccines by the National Strategic Committee, the campaign was implemented by the Vaccination section of the MOPH, along with the Hamad Medical Corporation (HMC; Doha, Qatar) and the Primary Health Care Corporation (PHCC; Doha, Qatar).

### 4.1. December 2020

**Figure 2:**
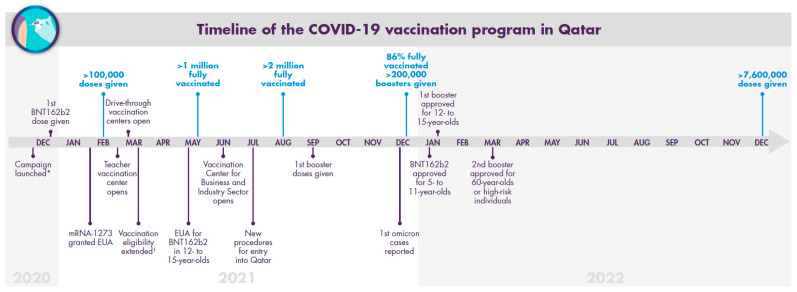
Chronology of the COVID-19 vaccination campaign in Qatar. * Frontline healthcare workers and those with severe or multiple chronic conditions or who were older than 70 years were prioritized. ^†^ People aged 50 years and older regardless of their health condition, individuals with moderate chronic medical conditions of any age, and additional healthcare providers and other key workers. EUA: emergency use authorization.

The Qatar COVID-19 vaccination program was launched in December 2020, after approval of BNT162b2 for emergency use by the MOPH [30]. After large-scale training of nurses and other staff to run mass vaccination sites and drive-through facilities [31], both BNT162b2 and mRNA-1273 provided the basis of Qatar’s vaccination campaign, which is described in detail below and summarized in Figure 2. The first BNT162b2 dose was administered on 23 December 2020 [32]. At this time, the alpha (B.1.1.7) and beta (B.1.351) variants were dominant in Qatar [33]. Vaccination of frontline healthcare workers, as well as individuals with severe or multiple chronic conditions and those older than 70 years, was initially prioritized [34]. Vaccination was later extended to sequentially younger age groups and select professions (e.g., teachers).

### 4.2. January–March 2021

By January 2021, thousands of individuals had received their first dose of the COVID-19 vaccine [35]. By mid-February, the MOPH granted emergency use authorization (EUA) to mRNA-1273 [36]. By this time, >100,000 vaccine doses had been administered and a vaccination center for teachers, school staff, and key workers opened [35,37]. In March 2021, Qatar began vaccinating people ≥50 years old regardless of their health condition, individuals with moderate chronic medical conditions of any age, and additional healthcare providers and other key workers in different ministries and government institutions [38]. Residents could also register for their vaccination via the MOPH website, with those meeting priority criteria immediately scheduled for vaccination, and those not yet meeting the criteria later contacted as soon as they became eligible [39]. To ensure eligible individuals were aware that they could receive the vaccine, they were contacted directly via text message or telephone to arrange for their vaccination appointment. In March, drive-through vaccination centers were opened in Lusail and Al Wakra [40].

### 4.3. April–June 2021

Beginning in April, the delta (B.1.617.2) variant became the predominant strain in Qatar, which was coincident with outbreaks in India and Nepal [33,41]. On May 10, the US Food and Drug Administration (FDA) expanded the EUA of BNT162b2 to include 12-to-15-year-olds based on the favorable safety profile, noninferior immune response compared with young adults, and the observed 100% vaccine efficacy against COVID-19 seen in the pivotal phase 3 trial [42,43]. Therefore, the MOPH included 12-to-15-year-old adolescents in the vaccination campaign from May 16 and used BNT162b2 only [44]. By the end of May, >1 million people in Qatar had been fully vaccinated [35]. To be considered fully vaccinated at this time, an individual must have received the last dose of an approved COVID-19 vaccine primary series. In June, the Qatar Vaccination Center for the Business and Industry Sector, which was one of the largest vaccination centers globally, was opened in Doha [45]. The center was a collaboration among several government, public, and private institutions and was created to vaccinate key business and industry workers [46]. With 700 staff and 300 stations, it had the capacity to administer >25,000 vaccine doses daily.

### 4.4. July–December 2021

In July, it was announced that all eligible individuals entering the country had to be fully vaccinated with vaccines approved by the MOPH [47]. Vaccines approved by the MOPH as of January 2022 were BNT162b2, mRNA-1273, ChAdOx1-SARS-CoV-2 (Vaxzevria/Covishield^TM^; AstraZeneca, Södertälje, Sweden), and Ad26.COV2.S (Janssen, Horsham, PA, USA) [48]. BBIBP-CorV (Sinopharm, Beijing, China), CoronaVac (Sinovac, Beijing, China), Gam-COVID-Vac (Sputnik V; Gamaleya Research Institute, Moscow, Russia), and BBV152 (Covaxin; Bharat Biotech, Hyderabad, Telangana, India) were conditionally approved [48]. Cases were dominated by the B.1.1.7-like and delta variants, but the delta variant decreased after travel restrictions were implemented [41]. By the end of August, >2 million residents had been fully vaccinated [44]. In September, the first booster dose of either BNT162b2 or mRNA-1273 was administered, with a focus on individuals most at risk of severe infection [49,50]. On December 17, the first four cases of the omicron (B.1.1.529) variant were reported in Qatar, all among nationals and residents returning from other countries [51]. By the end of 2021, >200,000 booster vaccination doses had been administered, with 86% of the population fully vaccinated with the initial 2-dose series [49,50].

### 4.5. January 2022–January 2023

**Figure 3:**
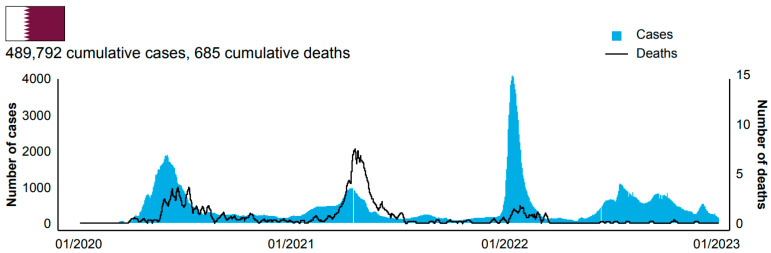
COVID-19 statistics in Qatar. Data and images are from the World Health Organization COVID dashboard (https://covid19.who.int/ [accessed on 4 January 2023]) and are current as of 3 January 2023.

In January 2022, BNT162b2 booster was approved in Qatar for use in adolescents 12–15 years of age, administered ≥6 months after the last dose of the primary series [52]. The BNT162b2 primary series was also approved for use in children aged 5–11 years [53]. In March 2022, a second booster (dose 4) of either BNT162b2 or mRNA-1273 was approved for adults ≥60 years old or any individual at high risk of severe COVID-19 infection, administered 4 months after dose 3 or COVID-19 infection [54]. The total number of vaccine doses administered as of 23 December 2022 is summarized in Table 1. By this time, more than 7.6 million vaccine doses had been administered, almost 73,000 of which were given to children 5–11 years of age. By 3 January 2023, Qatar had nearly 490,000 cumulative COVID-19 cases and 685 associated deaths (Figure 3).

## 5. Real-World Vaccine Effectiveness Data from Qatar

The COVID-19 vaccination program was supported by real-world effectiveness data, allowing public health responses to be implemented in a timely manner. Numerous reports from Qatar on the effectiveness of BNT162b2 and mRNA-1273 were identified, including effectiveness against emerging variants and in special populations [55,56,57,58,59,60,61,62,63,64,65]. It is worth noting that vaccine effectiveness was defined differently among studies; for example, it was often derived using the percentage reduction in infection hazard ratios or calculated as 1 minus the ratio of confirmed COVID-19 cases in the vaccine versus placebo groups [27,28,66]. Some of the findings of these studies are discussed in more detail below.

The rate of severe disease or death was shown to be approximately 3-fold lower among fully vaccinated individuals (i.e., received 2 doses of BNT162b2 from 23 December 2020–28 March 2021) who developed breakthrough infections than in matched unvaccinated individuals [67]. Increasing age, symptoms at baseline, and being unvaccinated were associated with severe disease. Moreover, in a large study conducted from 29 December 2020–10 May 2021, vaccine effectiveness against the alpha variant was shown to be 88.1% and 100% after 1 and 2 doses of mRNA-1273, respectively, and corresponding values against the beta variant were 61.3% and 96.4% [68]. The effectiveness against any severe, critical, or fatal disease overall was 81.6% after dose 1 and 95.7% after dose 2. Similarly, a test-negative case-control analysis of real-world data from the national COVID-19 databases demonstrated that estimated effectiveness after a single dose of the vaccine was 29.5% and 16.9%, respectively, whereas after dose 2, BNT162b2 was estimated to be 89.5% and 75.0% effective against infection with the alpha and beta variants, respectively, [69]. The effectiveness of BNT162b2 against any severe, critical, or fatal disease was 54.1% (1 dose) and 100.0% (2 doses) with the alpha variant, and 0% and 100.0%, respectively, with the beta variant. Regarding the delta variant, a large real-world analysis reported that 88 and 1126 infections occurred among those who received either 1 (*n* = 950,232) or 2 (*n* = 916,290) doses of BNT162b2, respectively, and 60 and 187 infections among those who received 1 (*n* = 564,468) or 2 (*n* = 509,322) doses of mRNA-1273, up to 7 September 2021 [70]. Despite the emergence of breakthrough infections, effectiveness against delta-related severe, critical, or fatal COVID-19 disease was 93.4–96.1% after dose 2 of either vaccine.

Real-world studies also provided useful information regarding the effectiveness of booster vaccination with BNT162b2 or mRNA-1273 against symptomatic SARS-CoV-2 infection and COVID-19–related hospitalizations and deaths during the omicron wave. In a matched retrospective cohort study using data from the national database of COVID-19 vaccination, laboratory testing, hospitalizations, and deaths (19 December 2021–26 January 2022), BNT162b2 and mRNA-1273 boosters were associated with 49.4% and 47.3% reductions in the incidence of symptomatic omicron infection, respectively, compared with the 2-dose primary series [71]. Few severe COVID-19 cases were observed either with or without a booster, confirming durability of protection against hospitalization and death up to several months after completion of the primary series. A similar study carried out through 28 February 2022, showed BNT162b2 booster effectiveness of 59.9% and 43.7% against symptomatic omicron BA.1 and BA.2 subvariants in the first month, respectively, followed by waning of protection in subsequent months, with similar findings for the mRNA-1273 booster [63]. Although vaccine protection was short lived against symptomatic omicron infections, mRNA vaccine effectiveness against omicron-associated hospitalization and death was high (>70%) and durable.

Real-world effectiveness data from Qatar were also available in special populations. Asymptomatic and symptomatic SARS-CoV-2 infections were reported in a study of 782 registered kidney transplant recipients, of whom 76.9% (*n* = 601) received ≥1 vaccine dose (mostly BNT162b2) by the end of the study (i.e., 21 July 2021, which corresponded to the alpha and beta variant waves in Qatar) [66]. Vaccine effectiveness was considerable in immunosuppressed kidney transplant recipients, with an estimated vaccine effectiveness against any severe, critical, or fatal COVID-19 disease of 85.0% at ≥42 days after dose 2. However, protection developed slowly and did not reach a high level until several weeks after dose 2. Additionally, in a study in pregnant women in a Qatar hospital between 20 December 2020 and 30 May 2021, mRNA vaccine effectiveness in preventing confirmed infection was estimated at 87.6% and 86.8% at ≥14 days after dose 2 in matched cohort (*n* = 407) and test-negative case-control (*n* = 255) analyses, respectively [64].

These real-world evidence studies helped support the COVID-19 vaccination program. Studies throughout the pandemic supported the benefit of full vaccination and boosters to protect against severe disease or death among both the general population [67,68,71] and special populations [64,66] in Qatar. They also helped officials to better understand the changing landscape of the pandemic and vaccine effectiveness as different variants emerged in Qatar [68,70,71]. These data helped support the key messages and policies put in place during the COVID-19 pandemic.

## 6. Previous Experience and Additional Factors Potentially Integral to the Qatar COVID-19 Vaccination Campaign

In the view of the authors of this review, several factors were likely integral to the successful implementation of the COVID-19 vaccination campaign in Qatar. For instance, the existence of the national seasonal influenza vaccination campaign meant that Qatar already had a well-established platform for vaccine delivery [72,73], ensuring swift implementation of the national COVID-19 vaccination campaign. Additionally, increased healthcare worker and vaccinator capacity was used to set up mass outreach centers, such as the Qatar National Convention Center, the Qatar Vaccination Center, and the Vaccination Center in the industrial area, catering to immigrant workers in Qatar and the drive-through centers.

In addition to the Adverse Event Following Immunization (AEFI) reporting system that was established in 1985 [74], the creation of a national database collating demographic data, SARS-CoV-2 testing results, and vaccination-related information permitted assessment of the effectiveness of the vaccination campaign [67] and enabled early notification and investigation of adverse events and regular feedback, resulting in better uptake of the COVID-19 vaccines. Furthermore, compliance with vaccination was generally high among people in Qatar [56,57,58,59]. Finally, the monitoring of wastage was easier. 

## 7. Vaccine Hesitancy and Mitigation of Misinformation

High vaccine uptake was critical to the success of COVID-19 vaccination in preventing severe disease, hospitalization, and death, decreasing burdens on healthcare systems and lowering the risk of new variants emerging [75]. Vaccine hesitancy, which is any refusal to be vaccinated or delay in acceptance of vaccination despite ready availability, is a complex issue influenced by confidence, convenience, and complacency [76].

Concerns about vaccine safety have been reported in Qatar, with vaccine hesitancy rates estimated at about 20% among the general population and adults ≥65 years of age [55,77]. Populations expressing concern regarding COVID vaccination included healthcare workers, the education sector, parents of young adolescents, and pregnant women (Table 2) [55,56,57,58,59]. Vaccine hesitancy was highest among Qataris of working age (43%) and those in the educational sector (37%) and lowest among the immigrant population (17%) and parents of young adolescents (18%). Individual reasons for hesitation regarding COVID-19 vaccination varied. Concerns about vaccine safety and efficacy, as well as disparity in the belief regarding protection afforded by natural exposure versus vaccination, are cited as common reasons for hesitancy both globally and in Qatar [55,56,57,59,78].

The requirement for booster doses of COVID-19 vaccinations (including duration of protection) also became a challenge. The need for booster doses was questioned by the public due to the breakthrough infections observed during the omicron surge in January 2022. People also questioned whether COVID-19 booster vaccinations would protect against future variants. Another major challenge was the hesitancy of parents to vaccinate their younger children [79,80].

A substantial part of the Qatari vaccination program focused on overcoming vaccine hesitancy. Qatar took an immediate stance to ensure that reliable sources of information were clarified [81], with the intent of mitigating misinformation. Within Qatar, the primary sources of reliable information included the MOPH, the HMC, the PHCC, the Ministry of Interior, and the Government Communications Office. Reliable international sources included the WHO, the US Centers for Disease Control and Prevention, the US FDA, and the European Center for Disease Prevention and Control. Additionally, a multimedia campaign targeting expatriates and their employers was used to increase awareness of COVID-19 and the preventive measures being taken [18]. Hesitancy was also addressed by interviews on television and radio by appointed individuals in multiple languages, thus aiding in disseminating information to migrant populations as well [82]. Authorized officials utilized social media platforms (e.g., Facebook, Instagram, Twitter, Snapchat) to answer queries raised by the public [83,84]. The government’s use of social media platforms may have helped mitigate circulation of misinformation, as a study conducted in Qatar from April to May 2021 found that 64.4% of university students or employees aged ≥18 years reported relying on social media as their primary source of COVID-19-related information [85]. Scientifically-backed information and frequently asked questions were posted on official websites [83]. As previously described, the Ehteraz smartphone application tracked viral transmission chains and provided accurate information [26]. It also provided current updates and statistics from the MOPH. Qatar’s well-established AEFI reporting platform confirmed that most adverse events were mild and resolved without many interventions, and that few severe AEFIs were reported [74]. This knowledge could have helped reduce hesitancy by allaying concerns regarding vaccine safety [86].

## 8. Assessing the Success of Qatar’s COVID-19 Campaign

The collective effects of COVID-19 public health measures, increasing vaccine uptake and support of the community, resulted in consistent decreases in the number of new infections in Qatar through the end of 2021 (Figure 3). Compared with other countries, and particularly those within the GCC, Qatar had one of the highest vaccination rates and one of the lowest case mortality rates (Table 3).

Implementation of Qatar’s COVID-19 response plan and vaccination program helped curtail the rapid spread of the virus observed at the outset of the pandemic, especially among craft and manual workers, who comprise a large portion of the population, are typically younger single men, and who often reside in densely crowded housing [15].

Reasons for the extremely low case mortality rates attributed to COVID-19 in Qatar are multifactorial. The young age of the population, with about 90% of the population 50 years and younger, is likely contributory [87]. Additionally, the involvement of the young and healthy population of craft and manual workers before the availability of vaccines might have also led to the low severity of disease observed in Qatar. Another factor is likely the country’s well-resourced healthcare system. Once vaccination was possible, the low mortality rates may have been sustained because of the vaccination program rapidly engaging high-risk populations, with 86% of the population fully vaccinated and >200,000 having received booster doses by the end of 2021 [49].

Like many countries, Qatar also had to contain the emergence of the highly transmissible omicron variant [88]. By January 2022, a wave of SARS-CoV-2 infections attributed to the omicron variant emerged in Qatar, predominantly affecting the unvaccinated and those who received their second primary dose >6 months before their first primary dose [89]. In response to the rising numbers of cases, Qatar and other GCC countries imposed measures such as online schooling, capacity limits, and vaccination requirements to attend public spaces (e.g., cinemas, theaters, and cultural events) [90].

## 9. Lessons Learned

As summarized in Table 4, several important challenges were identified during the COVID-19 campaign in Qatar and were successfully overcome.

Particular components of Qatar’s approach included having a national strategy for implementation, identifying priority groups for vaccination, and ensuring sufficient vaccine supply across the system to adequately target these groups. Another critical component was the role of the government in providing quarantine and isolation facilities [92], mandating use of the Ehteraz app to limit the spread of the disease [92], and cooperation with the private sector throughout the response [46].

Importantly, Qatar’s well-established vaccination plan enabled proactive and immediate implementation of the COVID-19 vaccination program as soon as vaccines became available. Although uptake of COVID-19 booster doses was initially challenging because of circulating misinformation, uptake eventually improved, with 66.0 persons boosted per 100 population as of 4 January 2023 [1]. The government’s orchestrated approach to mitigate fear and misinformation was instrumental in educating the population. Despite the approval of vaccination in younger children 5–11 years old in January 2022 [53], vaccination in this age group remains relatively low, with <73,000 doses administered as of 23 December 2022.

Importantly, whereas public safety measures were often based on symptomatic COVID-19 cases [93], an epidemiologic study of the first 5685 COVID-19 cases in Qatar (from February to April 2020) revealed that the majority (90.9%) of infected individuals had only mild symptoms or were asymptomatic [22]. These findings highlight the importance of COVID-19 testing, contact tracing, and vaccination, especially because the Qatar population can be at an increased risk of transmission due to mass gatherings or travel during religious holiday periods, a high-capacity international airport, and expatriates working or living in high-density areas [93]. In an effort to mitigate the virus spread, temporary isolation facilities were set up, including one of the largest COVID-19 isolation facilities specifically for asymptomatic or mildly symptomatic expatriate workers in Qatar [92].

Another challenge faced in Qatar was barriers to practicing the recommended COVID-19 preventive measures among migrant workers [91]. Interviews and focus group discussions among craft and manual workers from April to May 2020 showed that such barriers included language, diverse culture, knowledge and risk perception, health communications, and the workers’ lifestyle. These barriers may have potentially led to the initial rapid spread of COVID-19, especially among craft and manual workers, thus highlighting a particular need for effective and culturally sensitive health communication campaigns in Qatar. Finally, addressing the economic, social, and mental health aspects of the pandemic response may remain a challenge into the future.

## 10. Conclusions

A critical appraisal of Qatar’s approach to combatting COVID-19 is provided to better understand the successes and challenges in response to the COVID-19 pandemic. Qatar’s response strategy included engaging both the government and private sectors, limiting spread of the disease through the mandated use of the Ehteraz app, and providing quarantine facilities. Although a well-established vaccination plan was implemented as COVID-19 vaccines became available, and booster dose uptake is on the rise, vaccination among children aged 5–11 years is relatively low. Moreover, although public health measures often rely on symptomatic COVID-19 cases, a large proportion of cases in Qatar during the early pandemic were either mild or asymptomatic. To combat the spread of disease from such cases, Qatar implemented one of the largest quarantine facilities, which was designed specifically for asymptomatic or mildly symptomatic expatriate workers in the country. This narrative review illustrated the need for effective health communication, especially among migrant craft and manual workers. The key learnings from Qatar’s response to COVID-19 will be carried forward as a basis for any future national emergency responses in Qatar.

## Figures and Tables

**Table 1:** Total number of COVID-19 vaccines administered in Qatar as of 23 December 2022.

	Dose 1	Dose 2	Dose 3	Dose 4	Total
Pfizer	1,521,352	1,514,436	1,259,362	83,642	4,378,792
Moderna	1,132,198	1,083,948	624,023	211,391	3,051,560
AstraZeneca	94,597	41,359	17,399	17,115	170,470
Total	2,748,147	2,639,743	1,900,784	312,148	7,600,822

**Table 2:** Studies of COVID-19 vaccine hesitancy in Qatar.

	Migrant Majority [55]	Healthcare Workers [56]	Education Sector [57]	Parents of Young Adolescents [58]	Perinatal Women [59]
Study dates	15 October 2020–15 November 2020	15 October 2020–15 November 2020	February 2021	17 May 2021–3 June 2021	15 October 2020–15 November 2020
Population	Total population surveyed, 7821 Non-national participants, 6907	Healthcare workers, 1546	Qatar University employees and students, 462	Parents of 12-to-15-year-old adolescents, 4023	Pregnant or lactating women, 341
Study design	Cross-sectional survey	Cross-sectional survey	Cross-sectional survey	Cross-sectional study	Cross-sectional survey
Vaccine hesitancy rate	■ Qataris of working age, 43% ■ Immigrant population, 17%	■ Probably or definitely would not take the vaccine, 13% ■ Unsure, 25%	■ Not willing to be vaccinated, 37%	■ Overall, 18% ■ Highest in parents of 12-year-olds, 22% ■ Lowest in parents of 15-year-olds, 15%	■ Hesitant, 25% ■ Unsure, 26%
Concerns	■ Vaccine information trust deficit ■ Concerns about vaccine safety ■ Belief disparity between natural exposure vs. vaccination	■ Lack of trust in vaccine efficacy ■ Feel vaccine is not safe ■ Distrust of pharmaceutical companies ■ Belief disparity between natural exposure vs. vaccination	■ Vaccine efficacy and safety	■ Want to “wait and see”	■ Efficacy and safety ■ Belief disparity between natural exposure vs. vaccination ■ Belief that vaccines are promoted for financial gain
Proposed measures to address vaccine hesitancy	■ Use social media that the population reads to provide trustworthy education	■ Vaccine education	■ Continuous research to track factors influencing vaccine acceptance	■ Effective health promotion awareness campaigns	■ Endorsement by doctor, health service, the MOPH, or the WHO ■ Reading scientific research describing efficacy

MOPH: Ministry of Public Health; WHO: World Health Organization.

**Table 3:** COVID-19 cases, deaths, and vaccination rates globally and by select country.

	Cases, *n*	Deaths, *n* (%)	Fully Vaccinated, %
Global	655,689,115	6,671,624 (1.02)	64.5
Qatar	489,792	685 (0.14)	98.9
Bahrain	696,614	1536 (0.22)	72.1
Kuwait	662,747	2570 (0.39)	78.3
Oman	399,154	4628 (1.16)	59.7
Saudi Arabia	827,071	9521 (1.15)	72.8
United Arab Emirates	1,047,109	2348 (0.22)	99.0
United Kingdom	24,135,084	198,937 (0.82)	74.6
United States	99,423,758	1,082,265 (1.09)	68.3
South Africa	4,048,580	102,568 (2.53)	35.5
China	10,322,499	31,914 (0.31)	86.8

Data are from the World Health Organization COVID dashboard (https://covid19.who.int/ [accessed on 4 January 2023]). “Fully vaccinated” is based on receipt of the last dose of the primary series of approved COVID-19 vaccines in the specified regions.

**Table 4:** A summary of the challenges and successes of Qatar’s COVID-19 campaign.

Challenges	Successes
Protecting the health, well-being, and prosperity of Qatar’s citizens and residents by preparing for, monitoring, responding to, and recovering from COVID-19 outbreaks [24]	Development of the NRP enabled timely and informed decision-making; Creation of a cross-functional government/agency team to support the health sector, maintain essential and critical services, engage with the public, and maintain public confidence,
Access to healthcare due to rapid spread of COVID-19 among migrant workers [18,25]	Dedicated COVID-19 hospitals enabled care of patients with COVID-19 while maintaining routine care for other patients; Healthcare centers for migrant workers close to where they live, Provision of approximately 4000 acute and intensive-care beds, Rigid infection control measures for staff, patients, and visitors, Provision of PPE, Allocation of isolation and quarantine facilities, Expansion of available tele-health and tele-medicine services, Creation of multilingual tools to maximize outreach of public health messaging.
Limiting viral transmission [22,26,47,91]	Ehteraz app for smartphones (mandatory) to provide accurate information about viral transmission chains; Travel restrictions into and out of Qatar; Requirement that all people entering the country be fully vaccinated; Interviews and focus group discussions with migrant workers to identify barriers to practicing recommended preventive measures highlighting the need for effective and culturally sensitive health communications.
Vaccine availability [29,30,31,36,45,46,49,50,72,73]	Early procurement of COVID-19 vaccines and initiation of a national COVID-19 vaccination campaign; EUA granted for various vaccines by MOPH; Existing infrastructure, (i.e., a well-established platform for vaccine delivery) help with swift implementation of the national COVID-19 vaccination campaign; Large-scale training of nurses and other staff; Mass vaccination sites with drive-through facilities; Opening of the Qatar Vaccination Center for Business and Industry Sector (one of the largest vaccination centers in the world) to vaccinate key business and industry workers; Implemented a wide-reaching vaccination campaign.
Protection of at-risk populations [34,56,57,58,59,67,74]	Prioritization of vaccination of frontline healthcare workers, people with severe or multiple chronic conditions, and the elderly; Subsequent rollout to lower-risk populations; Creation of a national database collating demographic data, SARS-CoV-2 testing results, and vaccination-related information; Well-established AEFI reporting platform for early notification of AEs, early investigation, regular feedback, and better vaccine utilization.
Emerging variants [41,49,50,90]	Widespread vaccination of the population; Travel restrictions; Measures imposed (i.e., online schooling; capacity limits; required vaccination to attend cinemas, theaters, and cultural events) in response to surges in numbers of cases caused by variants.
Timely public health responses	Monitoring of real-world effectiveness data, particularly regarding emerging variants and special populations.
Vaccine hesitancy and misinformation [18,26,74,81,82,83,84]	Immediate stance by Qatar government to highlight reliable national and international sources of information; Multimedia campaign targeting expatriates and their employers to increase awareness of COVID-19 and preventive measures; Interviews on television and radio by appointed individuals in multiple languages; Use of social media platforms (e.g., Facebook, Instagram, Twitter, Snapchat) to clarify queries raised by the public; Ehteraz smartphone app tracked transmission; AEFI reporting platform permitted easy tracking of AEs after vaccination.
Measuring success of the vaccination program (see Table 3)	Comparison of vaccination rates with other countries revealed Qatar has one of the highest vaccination rates and one of the lowest case mortality rates.

AE: adverse event; AEFI: Adverse Event Following Immunization reporting system; EUA: emergency use authorization; MOPH: Ministry of Public Health; NRP: national response plan; PPE: personal protective equipment.

## Data Availability

No new data were created or analyzed in this study. Data sharing is not applicable to this article.
